# Identification and Validation of an Antivirulence Agent Targeting HlyU-Regulated Virulence in *Vibrio vulnificus*

**DOI:** 10.3389/fcimb.2018.00152

**Published:** 2018-05-11

**Authors:** Saba Imdad, Akhilesh Kumar Chaurasia, Kyeong Kyu Kim

**Affiliations:** Department of Molecular Cell Biology, Samsung Medical Center, Sungkyunkwan University School of Medicine, Suwon, South Korea

**Keywords:** *Vibrio vulnificus*, *rtxA1*, hemolysin, *hlyU*, drug identification and repositioning, fursultiamine hydrochloride

## Abstract

Antimicrobial resistance (AMR) in pathogens is the result of indiscriminate use of antibiotics and consequent metabolic/genetic modulation to evolve survival strategies and clonal-selection in AMR strains. As an alternative to antibiotic treatment, antivirulence strategies are being developed, not only to combat bacterial pathogenesis, but also to avoid emerging antibiotic resistance. *Vibrio vulnificus* is a foodborne pathogen that causes gastroenteritis, necrotizing wound infections, and sepsis with a high rate of mortality. Here, we developed an inhibitor-screening reporter platform to target HlyU, a master transcriptional regulator of virulence factors in *V. vulnificus* by assessing *rtxA1* transcription under its control. The inhibitor-screening platform includes wild type and Δ*hlyU* mutant strains of *V. vulnificus* harboring the reporter construct P_*rtxA*1_*::luxCDABE* for desired luminescence signal detection and control background luminescence, respectively. Using the inhibitor-screening platform, we identified a small molecule, fursultiamine hydrochloride (FTH), that inhibits the transcription of the highly invasive repeat-in-toxin (*rtxA1*) and hemolysin (*vvhA*) along with other HlyU regulated virulence genes. FTH has no cytotoxic effects on either host cells or pathogen at the tested concentrations. FTH rescues host cells from the necrotic cell-death induced by RtxA1 and decreases the hemolytic activity under *in vitro* conditions. The most important point is that FTH treatment does not induce the antivirulence resistance. Current study validated the antivirulence strategy targeting the HlyU virulence transcription factor and toxin-network of *V. vulnificus* and demonstrated that FTH, exhibits a potential to inhibit the pathogenesis of deadly, opportunistic human pathogen, *V. vulnificus* without inducing AMR.

## Introduction

*Vibrio vulnificus* is an opportunistic, Gram-negative, halophilic pathogen (Starks et al., 2000). It causes necrotizing wound infections, cellulitis, gastroenteritis, and devastating septicemia, with mortality rates up to 50%, especially in immunocompromised subjects (Linkous and Oliver, 1999; Jones and Oliver, 2009), which is one of the highest among foodborne diseases (Mead et al., 1999). *V. vulnificus* easily evades the host innate immune system to quickly propagate in the bloodstream, which causes death within a few days of infection. This fast rate of disease progression is attributed to the presence of capsular polysaccharide and repeat-in-toxin (RtxA1), which are both suggested to be inhibitory factors of phagocytosis (Tamplin et al., 1985; Lo et al., 2011). The source of *V. vulnificus* infection is usually raw and undercooked seafood (Gulig et al., 2005). Third or fourth generation cephalosporins, tetracycline (Chiang and Chuang, 2003; Lee et al., 2014), and quinolones can be used to kill *V. vulnificus* during infection (Tang et al., 2002; Wong et al., 2015). However, recently, emergence of antimicrobial resistance (AMR) in various *Vibrio* species, including *V. parahaemolyticus* and *V. vulnificus*, has been reported in several countries (Baker-Austin et al., 2009; Elmahdi et al., 2016; Siboni et al., 2016; Baker-Austin and Oliver, 2018). Thus, *V. vulnificus*, an antimicrobial resistant, deadly, opportunistic human pathogen is prevalent in a spatiotemporal manner in estuaries and is considered to be an environmental and clinical burden, posing a major health concern among the foodborne infectious diseases (Siboni et al., 2016; Heng et al., 2017).

AMR in general, is the mixed response of a pathogen's offensive and defensive survival strategies against the stress imposed by antimicrobial agents. Consequently, AMR strains continue to emerge through the clonal selection of mutationally altered variants during antibiotic treatment (Blair et al., 2015). Moreover, antibiotic treatment kills bacterial pathogens in the bloodstream, leading to the release of cytotoxins, and lipopolysaccharide (LPS) (Jackson and Kropp, 1992; Prins et al., 1994). This in turn can cause a hyper-immune response, toxic anaphylactic shock, and fatality for patients (Opal, 2010). Therefore, virulence specific therapeutics are being evolved as an alternative approach to circumvent both AMR and pathogenesis. Antivirulence therapeutic strategy may prove advantageous because (**a**) it may not induce a pathogen to develop AMR (**b**) being non-antibiotic in nature, it may not disturb the native gut-microbiota, and (**c**) it acts solely by inhibiting virulence factors without threatening general physiology and survival of pathogen (Cegelski et al., 2008).

*In vivo*-induced antigen technology (IVIAT) in *V. vulnificus* has shown that virulence regulator HlyU is preferentially induced during infection conditions (Kim et al., 2003). Among the virulence factors contributing toward *V. vulnificus* pathogenesis, toxins are considered to be major players in the progression of pathogenesis and evasion of the host innate immune system (Lee et al., 2004, 2008a,b; Kim et al., 2008; Liu and Crosa, 2012; Letchumanan et al., 2017). HlyU is a master transcriptional regulator of virulence in *V. vulnificus*, that has evolved from the ArsR/SmtB family (Busenlehner et al., 2003; Saha and Chakrabarti, 2006). It positively regulates the expression of the major pore-forming toxins (PFT) in *V. vulnificus*, RtxA1, and hemolysin (VvhA) (Liu and Crosa, 2012). HlyU regulates the transcription of *rtxA1* by direct binding to the upstream regulatory region of *rtxA1* promoter (Liu et al., 2009, 2011; Liu and Crosa, 2012). RtxA1-deficient strains are defective in host infection and increase the LD50 in mouse models by 100-fold (Kim et al., 2008; Shao et al., 2011). Microarray analysis revealed that *hlyU* positively regulates the *rtxA1* gene expression (Liu et al., 2007). HlyU has been reported to bind to the AT-rich upstream regulatory region at −417 to −376 bp of the *rtx* operon transcription start site, which codes for the major pore-forming toxin (PFT), RtxA1 during *V. vulnificus* infection (Liu et al., 2009, 2011; Liu and Crosa, 2012). Another key gene in the cytotoxin regulatory circuit is *hns*, which codes for a global repressor, H-NS. H-NS binds to five sites in the upstream regulatory region of *rtxA1* promoter (P_*rtxA*1_) and forms a bridge within DNA. As a result, it obstructs the movement or exclude the entry of RNA polymerase leading to the repression of *rtxA1* (Liu et al., 2009; Liu and Crosa, 2012) under non-pathogenic or free-living conditions. HlyU acts as anti-repressor of H-NS and binds to P_*rtxA*1_ with higher affinity than H-NS; thereby, de-repressing *rtxA1* gene expression (Liu et al., 2009). The expression of *hlyU* is regulated by quorum sensing master regulator, SmcR, a LuxR homolog of *V. harveyi* (Shao et al., 2011; Liu and Crosa, 2012). Therefore, identification of small molecules to inhibit the HlyU-controlled expression of virulence factors during host-pathogen interactions appears to be a robust strategy to tackle *V. vulnificus* virulence. In a previous study, a small molecule, resveratrol was identified using a host cell viability assay. Resveratrol inhibits the expression of *rtxA1* gene, whereas the cognate upstream regulator (HlyU) and VvhA were not found to be inhibited (Kim et al., 2010). To the best of our knowledge, there are no reports identifying a small molecule inhibitor targeting HlyU transcription factor and its cytotoxins to inhibit *V. vulnificus* virulence.

In this study, we targeted HlyU-regulated virulence factors by employing an antivirulence approach. We designed an inhibitor-screening platform composed of wild type and a Δ*hlyU* deletion mutant of *V. vulnificus* harboring *luxCDABE* under the P_rtxA1_ promoter. By screening 1840 small molecules comprising natural compounds and the FDA-approved Prestwick Library, we identified a non-toxic small molecule, *f* ursul*t*iamine *h*ydrochloride (FTH). We showed that FTH inhibits HlyU-regulated toxin genes (*rtxA1* and *vvhA*) at the transcriptional level without affecting the expression of *hns*, which acts as repressor of *rtxA1* (Liu et al., 2009). FTH does not inhibit the transcription of *hlyU*, which we tested using qRT-PCR and a transcriptional fusion of P_*hlyU*__*hlyU*-*luxCDABE*. The *luxCDABE* reporter-gene tag and its comparative expression under HlyU-regulated P_*rtxA*1_ and the non-specific synthetic P_*EM*7_ promoter in wild type cells and a Δ*hlyU* mutant revalidated the specificity of the reporter system targeting HlyU. Furthermore, treatment of FTH in wild type *V. vulnificus* harboring P_*rtxA*1_ or the synthetic P_*EM*7_ promoter showed a specific inhibition of luminescence with the P_*rtxA*1_ promoter, but not with the P_*EM*7_ construct, demonstrating that FTH specifically targets native chromosomal HlyU under ambient bacterial growth conditions. Being a thiamine derivative, FTH neither affects bacterial viability at the tested concentrations nor poses any toxicity to host cells under *in vitro* conditions. FTH also rescues HeLa cells from cytoskeleton destabilization and subsequent necrotic cell death induced by RtxA1 under *in vitro* conditions. The current study demonstrates that FTH can effectively inhibit the HlyU-regulated virulence factors RtxA1, VvhA, and *plpA*_2_ at the transcriptional level, and thus significantly reduced the virulence of *V. vulnificus* by disarming its powerful pore-forming toxins and its ability to kill host cells.

## Material and methods

### Bacterial strains and cell culture conditions

The strains and plasmids used in this study are listed in Table S1. *Vibrio vulnificus* MO6-24/O wild type (Wright et al., 1990) (hereafter termed as WT *V. vulnificus*), deletion mutants Δ*hlyU* (ZW141) and Δ*rtxA1* (MW064) of *V. vulnificus* MO6-24/O were used in this study (Lee et al., 2007; Jang et al., 2017). *V. vulnificus* was revived on a *Vibrio sp*. selective medium, Thiosulfate citrate bile salts sucrose—Oxoid (TCBS) agar plate. A single colony from TCBS-agar plate was inoculated in Luria-Bertani medium supplemented with 2% sodium chloride (LBS) broth and incubated at 37°C under orbital shaking culture conditions. The growth of *V. vulnificus* was measured by optical density at 600 nm (OD_600_) and enumerated by determining the bacterial colony forming units (CFU) on LBS-agar (1.5% agar) plate. The antibiotic concentration used for the selection and/or growth of recombinant and mutant strains was 2 μg/ml and 300 μg/ml for chloramphenicol (Cm) and kanamycin (Km), respectively. *E. coli* DH5α was grown in LB medium (broth/agar) with the appropriate concentrations of antibiotics (ampicillin, Amp: 100 μg/ml and chloramphenicol, Cm: 33 μg/ml) to select recombinant strains harboring plasmids with corresponding antibiotic resistance genes. Unless stated otherwise, HeLa cells were cultured at 37°C with 5% CO_2_ in Dulbecco's Modified Eagle's Medium (DMEM) with high glucose, containing 10% fetal bovine serum (FBS) for routine culturing.

### Construction of inhibitor-screening reporter strain, non-specific promoter-driven luminescent strain, and *HlyU::luxCDABE* transcriptional fusion strain in *V. vulnificus*

The promoter-less empty backbone plasmid pBBRMCS2*::luxCDABE* (Lenz et al., 2004) was used to construct the reporter strain and other genetically engineered strains. The backbone plasmid, pBBRMCS2*::luxCDABE* was kindly provided by Prof. Sang Ho Choi (Seoul National University, Korea). *V. vulnificus* genomic DNA was extracted using G-Spin genomic DNA extraction kit (Intron Biotech, Korea). High-fidelity Taq DNA polymerase was used for PCR amplification. The restriction endonuclease enzymes were purchased from New England Biolab (NEB, USA). Plasmid extraction (Intron Biotech, Korea) and gel purification (Cosmogenetech LaboPass Gel Extraction kit, Korea) were performed as per the manufacturers' protocols. All recombinant DNA techniques were performed according to the *Standard Laboratory Manual* (Sambrook et al., 1989). Briefly, the 754 bp promoter region of *rtxA1* (P_*rtxA*1_) in *V. vulnificus* was PCR-amplified from genomic DNA using specific primers (Table S2). The gel-purified P_*rtxA*1_ PCR product and pBBRMC2*::luxCDABE* plasmid were restriction digested with SacI and SpeI, and ligated and cloned at the same sites of the promoter-less pBBRMC2::*luxCDABE* vector to achieve the pBBRMC2_P_*rtxA*1_::*luxCDABE* reporter plasmid.

To make a transcriptional fusion of *hlyU* with *luxCDABE*, the *hlyU* ORF along with its 128 bp native promoter was amplified using specific primer pairs (Table S2). The resulting 425 bp PCR product and the vector pBBRMCS2*::luxCDABE* were restriction digested with SacI and BamHI. The restriction-digested vector and inserts were gel-purified and ligated at the same sites of the promoter-less pBBRMCS2*::luxCDABE* plasmid to generate the transcriptional fusion plasmid pBBRMCS2_P_*hlyU*_*_hlyU-luxCDABE*. A 150 bp DNA fragment containing the 47 bp P_*EM*7_ synthetic promoter was amplified from pGEN-*luxCDABE* (Lane et al., 2007) using the specific primer pairs shown in Table S2. The P_*EM*7_ DNA fragment and the pBBRMCS2::*luxCDABE* vector were restriction digested with SacI and BamHI. The 150 bp DNA fragment containing the P_*EM*7_ promoter possessed an internal BamH1 site, resulting in an 89 bp digested P_*EM*7_ fragment, containing the complete synthetic promoter. The SacI-BamHI digested vector and the 89 bp P_*EM*7_ promoter fragment were ligated and cloned directionally to obtain the pBBRMCS2_P_*EM*7_*::luxCDABE* plasmid.

The putative clones obtained were confirmed by plasmid isolation, followed by restriction digestion and analytical agarose gel electrophoresis. Both strands of all cloned DNA fragments were sequenced by the di-deoxy method, and the accuracy of sequences was ascertained by BLAST and peak analysis of nucleotides. The recombinant plasmids and the empty vector backbone (pBBRMCS2::*luxCDABE*) were electroporated into WT and *hlyU* deletion mutant, Δ*hlyU* (Table S1) of *V. vulnificus* as described elsewhere (Klevanskaa et al., 2014), with slight modification. Briefly, 50 ml cultures were grown from single colonies picked from TCBS agar. The cells were allowed to grow up to OD_600_ 0.8 equivalent cells. Cells were harvested by centrifugation at 4000 rpm for 15 min at 4°C. All the steps, buffers, media, and cuvettes during the electrocompetent cell preparation were maintained at 4°C or on ice. The cell pellet was washed with 50 ml cold 1 mM Tris-Cl buffer (pH 6.0) supplemented with 200 mM sucrose. After washing, the cells were resuspended in 1000 μl cold GYT medium (10% glycerol, 0.125% w/v yeast extract, and 0.25% w/v tryptone). An aliquot of 100 μl of electrocompetent cells was added to a chilled electroporation cuvette with a 0.1 cm electrode gap. Approximately 700 ng of plasmid DNA was added to 100 μl of electrocompetent cells just before the electroporation pulse. The cells were electroporated using the BIORAD Gene Pulser X-cell instrument at 750 V, 25 μF capacitance, and 200 Ω resistance. The electroporated cells were recovered in 900 μl LB for 1 h and plated on LBS-plates supplemented with 2 μg/ml Cm. By this approach, *V. vulnificus* WT strain harboring pBBRMC2_P_*rtxA*1_::*luxCDABE* (hereafter termed as WT reporter strain) under the regulation of native chromosomal *hlyU* was created for screening the chemical libraries. The *V. vulnificu*s Δ*hlyU* reporter strain harboring pBBRMC2_P_*rtxA*1_::*luxCDABE* was used as a control to assess the background signal.

### Screening of chemical libraries

Overnight cultures of WT and Δ*hlyU* (control) reporter strains were sub-cultured with 1% inoculum and supplemented with 2 μg/ml chloramphenicol in LBS for primary chemical screening. A total number of 1,840 small molecules (consisting of 800 natural product compounds and 1,040 Food and Drug Administration [FDA]-approved chemicals from the Prestwick Library) were screened. The final concentration of tested chemicals for screening was 20 μM. The luminescence and bacterial growth (OD_600_) of samples were recorded in multi-well plates after 6 h using a microplate reader (Tecan Infinite M200, Switzerland). The primary hits were identified on the basis of reduction in the luminescence signal per unit OD_600_ relative to the untreated or vehicle (DMSO 0.2% v/v)-treated control. The hits were re-checked in a concentration-dependent manner, and were sorted for further analysis. The selected chemicals were purchased from Prestwick Chemicals, and stocks of 10 mM or 100 mM were prepared in DMSO or water.

### Quantitative real-time PCR

Overnight cultures from freshly streaked single colonies of WT or Δ*hlyU V. vulnificus* were diluted to 2 × 10^6^ CFU/ml in 3 ml fresh LBS and incubated at 37°C, 220 rpm in 14 ml polypropylene culture tubes. WT *V. vulnificus* was treated with the small molecule inhibitors or with DMSO (as a control) and was grown up to OD_600_ ~1.8–2.0. RNA isolation from *V. vulnificus* reporter strains was carried out with the similar bacterial cells input and incubated for 9 h in 48-well plates with or without FTH. The treated cells were harvested by centrifugation at 5000 × g for 5 min. The cell pellets devoid of supernatant were immediately treated with RNA-Protect reagent (QIAGEN). Total RNA was isolated using the QIAGEN RNeasy Mini kit as per the manufacturer's instructions. A total of 1 μg of RNA from both the control and treated samples was subjected to DNase I treatment (amplification grade; Sigma) for 15 min at room temperature. The reaction was stopped by adding stop buffer and DNaseI was heat inactivated by incubating the samples at 70°C for 10 min. First-strand cDNA synthesis was performed using the Ecodry Premix (random hexamer) kit (Takara Bio, USA). DNase I-treated RNA samples were subjected to target gene PCR amplification to check for genomic DNA contamination. The primers (Table S2) used for quantitative RT-PCR (qRT-PCR) ranged from 19 to 21 bp in length with annealing temperatures of 55 ± 2°C, and the amplified product size of 200 bp. Each qRT-PCR reaction consisted of 300 nM forward and reverse primers, 100 ng cDNA, and 1X Taq universal SYBR Green supermix (Bio-Rad) containing dNTPs and Taq polymerase with its buffer. Expression of the *rtxA1, vvhA, hlyU*, and *hns* genes was normalized with the endogenous *gyrB* gene (Table S2). Relative gene expression was analyzed using the 2^−ΔΔCT^ method (Schmittgen and Livak, 2008). Three independent qRT-PCR experiments were performed for the analysis. Statistical significance was calculated by student's *t*-test (*p* < 0.05).

### Host cell viability assay

A host cell viability assay as a measure of cell proliferation was performed to evaluate the cytotoxicity of the FTH small molecule using the EZ Cytox cell viability assay kit (DoGen, Korea). HeLa cells were seeded at a density of 2 × 10^4^ cells per well in 100 μl of culture medium using 96-well plates. After 24 h of incubation, FTH was added at concentrations ranging from 1 to 2048 μM at two-fold increments. After 48 h of drug incubation, EZ Cytox kit solution was added to each well and plates were incubated for 2 h. The absorbance of dye was measured spectrophotometrically at A_450_ using a multi-plate reader (Tecan Infinite M200, Switzerland). The mammalian cell culture media was used as blank. The IC_50_ was calculated using GraphPad Prism 6.01. The data were plotted, transformed to log values, and then fitted onto a non-linear curve after normalization. FTH untreated cells were considered to be 100% viable.

### Hemolysis assay

Overnight cultures of WT *V. vulnificus* were inoculated in fresh LBS with or without FTH inhibitor (10–60 μM), and incubated for 3 h wherein Δ*hlyU* served as a positive control of hemolysis inhibition. The cell free culture supernatants were withdrawn and mixed with 1% human red blood cells (hRBCs) and were incubated at 37°C for 1 h under shaking culture conditions. The cell debris was removed by centrifugation at 1,500 rpm (0.2 × g). The hemolysis was visualized using absorption spectra (500–650 nm) with 1:1 dilution of supernatant to hRBCs. To calculate the hemolytic unit, the culture supernatants were diluted by 1/2, 1/4, and 1/8 with PBS and the remaining method was followed as described above. The hemolytic unit was expressed as the reciprocal of the dilution factor showing 50% hemolysis absorbance at 540 nm (A_540_) as described by Lee et al. (2013).

### Cytoskeleton staining and cellular phenotype

HeLa cells (3 × 10^4^) were cultured in 20 mm glass-bottom cell culture confocal discs for 48 h. The cells were replenished with fresh DMEM media without FBS before starting the experiment. HeLa cells were infected with 20 *moi* of WT *V. vulnificus* with or without the simultaneous addition of FTH (100 and 200 μM) and incubated for 1 h. HeLa cells were infected with 20 *moi* of Δ*hlyU* and Δ*rtxA1* strains of *V. vulnificus* (Table S1) as controls. Following incubation, the cells were washed with PBS, fixed with 4% paraformaldehyde (in PBS), and permeabilized with 0.1% Triton X-100 (in PBS). The cells were stained with 1 unit of rhodamine phalloidin (Invitrogen, USA) for 1 h in the dark (Kim et al., 2008). The cells were then washed with PBS and counterstained with 4′,6′-diamino-2-phenylindole (DAPI) for 5 min, followed by thorough PBS washing. The stained cells were imaged at 400 × magnification using a laser scanning confocal microscope (LSM 710, Zeiss). The frequency of cytoskeleton-destabilized rounded cells was obtained by counting 1000 cells in various microscopic fields of each sample. A control experiment was performed to assess the effect of FTH on WT *V. vulnificus* in DMEM medium. The LBS grown log phase culture of WT *V. vulnificus* cells was washed with PBS and 10^6^ equivalent bacterial cells were adjusted by measuring the optical density. The washed cells (10^6^) were incubated with 0, 100, and 200 μM FTH for an hour in DMEM medium. After incubation, the cells were harvested, washed with PBS, diluted, and plated on LBS agar plates to enumerate the CFU.

### Assessment of laboratory-induced adaptive evolution of antimicrobial resistance vs. antivirulence resistance

Fluoroquinolone antibiotics are one of the current successful therapeutic options for treatment of *V. vulnificus* infection. Norfloxacin was taken as a representative of the flouroquinolone antibiotics and compared with the antivirulent FTH for the adaptive evolution of their corresponding resistance against WT *V. vulnificus*. Norfloxacin was tested at the concentration of 0.155 μg/ml while FTH was tested at two different concentrations (40 and 60 μM), to evaluate antivirulence resistance. The norfloxacin concentration was selected by taking the average of a range of MIC_90_ values (0.063–0.25 μg/ml) for various *V. vulnificus* strains, as reported elsewhere (Morris et al., 1985). WT *V. vulnificus* culture was grown in LBS overnight and 1% inoculum was used for norfloxacin or FTH exposure and subsequent transfers for around 2 weeks. The treated and untreated *V. vulnificus* cultures were continuously transferred to observe the adaptive evolution of antimicrobial and antivirulence resistance in response to sustained antibiotic or antivirulence chemical pressure on WT *V. vulnificus*. In the case of norfloxacin-treated samples, the total input inoculum (1%) was centrifuged and resuspended in a new tube containing fresh LBS supplemented with 0.155 μg/ml norfloxacin. In the case of untreated or FTH-treated samples, 1% of 18 h grown culture was sub-cultured for next transfer. The sub-culturing and transfers were continued until bacterial growth was visible in norfloxacin treated culture. The MIC assay was performed according to CLSI guidelines (2012) by the microbroth dilution method in Mueller Hinton Broth (MHB) (Wiegand et al., 2008).

## Results

### HlyU-regulated transcription of virulence factors and the construction of inhibitor-screening reporter

**Figure 1:**
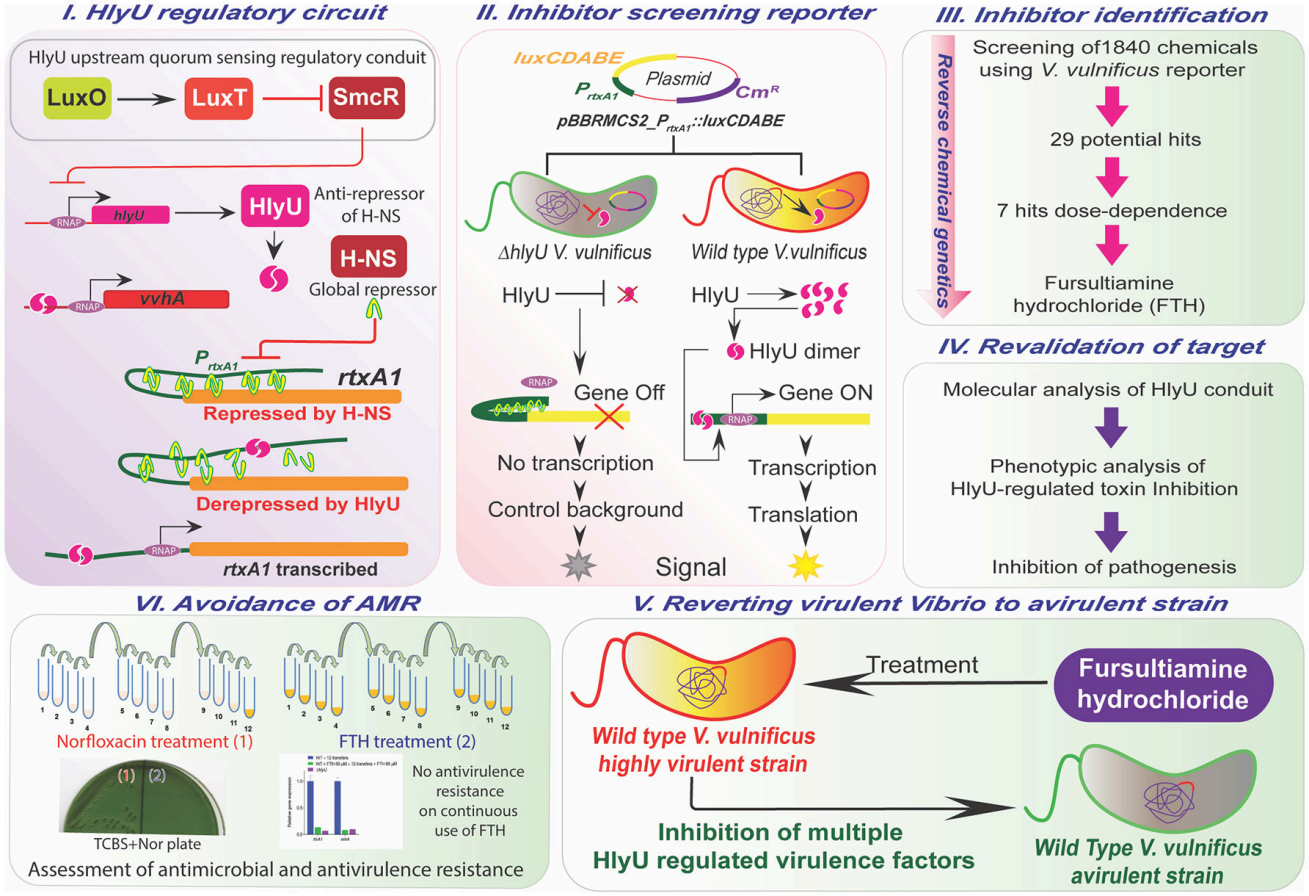
Overview of the study. The upstream and downstream regulatory network of HlyU **(Panel I)**, the design of the reporter platform **(Panel II)**, the workflow for the identification, validation, and assessment of antivirulence characteristics of small molecule inhibitor, FTH targeting HlyU in *V. vulnificus* **(Panels III–V)**, and adaptive evolution demonstrating the avoidance of antimicrobial and antivirulence resistance development during antivirulence approach **(Panel VI)**.

HlyU acts as master virulence transcription factor controlling an array of virulence genes including highly invasive toxins (Liu et al., 2007). Therefore, it is conceivable that HlyU is an important target for antivirulence therapeutics. An overview of the HlyU upstream and downstream regulatory cascades are depicted in (Figure 1I). Based on the *hlyU* regulatory cascades (Figure 1I), the scorable inhibitor-screening reporter platform was developed in *V. vulnificus* to identify inhibitors of the HlyU-regulated transcriptional network (Figure 1II). The reporter plasmid pBBRMC2_P_*rtxA*1_::*luxCDABE* was introduced into WT and Δ*hlyU V. vulnificus* strains (Figure 1II, Table S1). The reporter system was validated by measuring its luminescence at different time points, and the WT reporter strain showed maximum luminescence at 6 h of incubation in microtitre plates when inoculated with 1% overnight-grown culture (Figure S1). The Δ*hlyU* reporter strain luminescence was one-third of the WT reporter at the same time point. The Δ*hlyU* reporter strain therefore served as a background control and facilitated in identifying potential hits during the primary inhibitor screening (Figure 1II).

### Screening of chemicals for identifying inhibitor hits

**Figure 2:**
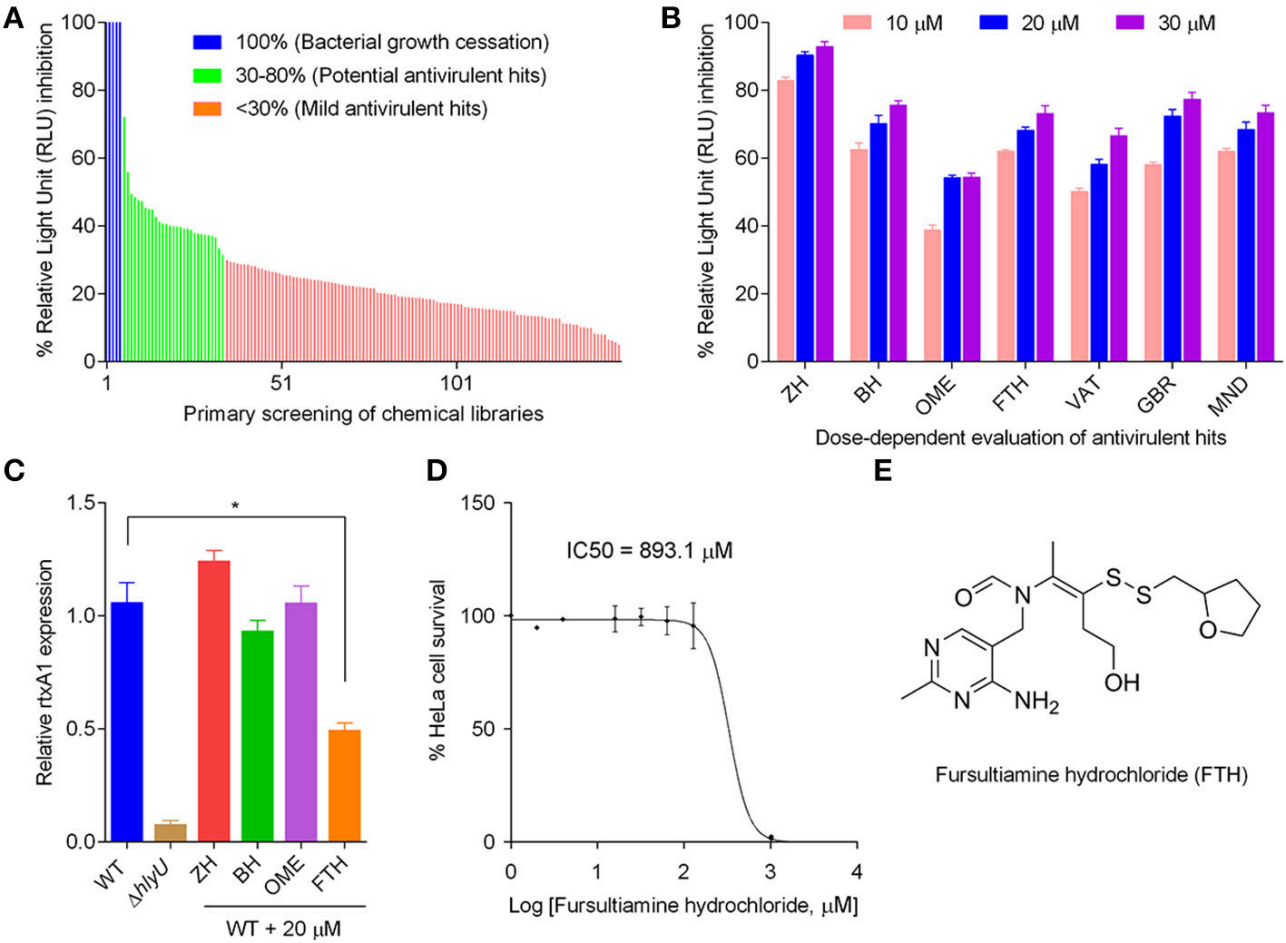
Steps to identify the antivirulence agent fursultiamine hydrochloride (FTH). **(A)** Primary screening of small molecule libraries (1,840 chemicals at 20 μM) using *V. vulnificus* reporter. A total of 150 chemicals were shown as representative for inhibition of relative light units (RLU) per unit optical density (OD_600_) after 6 h of incubation. The RLU/OD_600_ ratio and the percent inhibition of normalized luminescence signal allowed subdivision of chemicals into four groups. Complete (100%) RLU inhibition was observed with chemicals that allowed no or minimal growth of bacteria (“bacterial growth cessation”), and these were excluded from further analysis. The group showing more than 30% RLU inhibition is labeled “potential antivirulence hits.” **(B)** Dose-dependent response of selected chemicals from the group labeled “potential antivirulence hits” using *V. vulnificus* reporter. ZH, zuclopenthixol hydrochloride; BH, benzamil hydrochloride; OME, omeprazole; FTH, fursultiamine hydrochloride; VAT, vatalanib; GBR, GBR 12909 dihydrochloride; and MND, mebhydroline 1,5-naphtalenedisulfonate. **(C)** Quantitative RT-PCR of *rtxA1* gene of WT *V. vulnificus* following the treatment with ZH, BH, OME, and FTH (20 μM), which were selected because these chemicals did not inhibit *V. vulnificus* growth. **(D)** HeLa cell proliferation as a measure of the *in vitro* cytotoxicity of FTH using EZ-Cytox cell viability kit. A_450_ values were measured after 48 h, and IC_50_ values were calculated with GraphPad prism 6.01. **(E)** Structure of fursultiamine hydrochloride.

The primary screening was performed using the chemicals in the libraries at 20 μM concentration in the WT inhibitor-screening reporter strain (Figure 1III). The initial hits were grouped into three categories based on percent inhibition of WT reporter luminescence per unit OD_600_. Chemicals showing less than 30% inhibition were considered “*mild antivirulence hits,”* while chemicals showing more than 30% inhibition were categorized as “*potential antivirulence hits”* (29 chemicals) and were selected for further scrutiny. Chemicals showing 100% inhibition of luminescence signal due to cessation of bacterial growth were not the focus of this study (Figure 2A). Further evaluation of “*potential antivirulence hits”* based on the dose-dependent decrease in luminescence per unit OD_600_ narrowed the focus to seven molecules, namely zuclopenthixol hydrochloride (ZH), benzamil hydrochloride (BH), omeprazole (OME), fursultiamine hydrochloride (FTH), vatalanib (VAT), GBR 12909 dihydrochloride (GBR), and mebhydroline 1,5-naphtalenedisulfonate (MND). These chemicals showed a concentration-dependent inhibition of the luminescence signal in the WT reporter strain (Figure 2B). These seven chemicals were then subjected to careful examination regarding their effects on WT *V. vulnificus* growth pattern. The selected chemical treatments were compared with the DMSO placebo treatment in WT *V. vulnificus*, and growth patterns were monitored at OD_600_ every hour for 8 h. VAT, GBR, and MND were excluded because they affected *V. vulnificus* growth profile in a dose-dependent manner (data not shown). The remaining four chemicals were assessed for their effects on *rtxA1* mRNA expression levels in WT *V. vulnificus* at 20 μM concentration that was used for primary screening. RtxA1 gene expression test was considered the first key step toward finding a true inhibitor, since the reporter system was designed with the promoter of *rtxA1*, which is directly regulated by HlyU. The molecular analysis for relative *rtxA1* gene expression showed that only FTH significantly decreased *rtxA1* transcription (i.e., by at least 1.5-fold) when compared to the corresponding DMSO-treated control. The *rtxA1* mRNA level was negligible in Δ*hlyU*, which confirmed that HlyU is the key regulator of the major cytotoxin RtxA1 in *V. vulnificus* (Figure 2C). Moreover, the varying concentrations of FTH (20–60 μM and 100–200 μM) did not inhibit the growth, as also represented by the CFU counts of WT *V. vulnificus* (Figures S2, S3). One of the most important parameters for evaluating a potent candidate as a therapeutic agent is its toxicity to host cell or safety. FTH cytotoxicity was therefore evaluated using HeLa cells. FTH showed negligible toxicity toward the host cells since the IC_50_ value of FTH was found to be 893.1 μM (Figure 2D). The FTH concentration tested against *V. vulnificus* is 60–200 μM which is significantly lower than the IC_50_ concentration for the host cells. Therefore, FTH appeared to have the properties suitable for being developed as an antivirulence agent because it did not pose any toxicity toward host or pathogen in the range of concentrations used to inhibit the major cytotoxins RtxA1 and VvhA. FTH is the synthetic counterpart and active form of allithiamine, which occurs naturally in garlic. The disulfide bond in FTH is considered to be essential for its biological activity in treating thiamine deficiency (Lonsdale, 2004) (Figure 2E).

### Verification of the transcriptional conduits of HlyU by FTH

**Figure 3:**
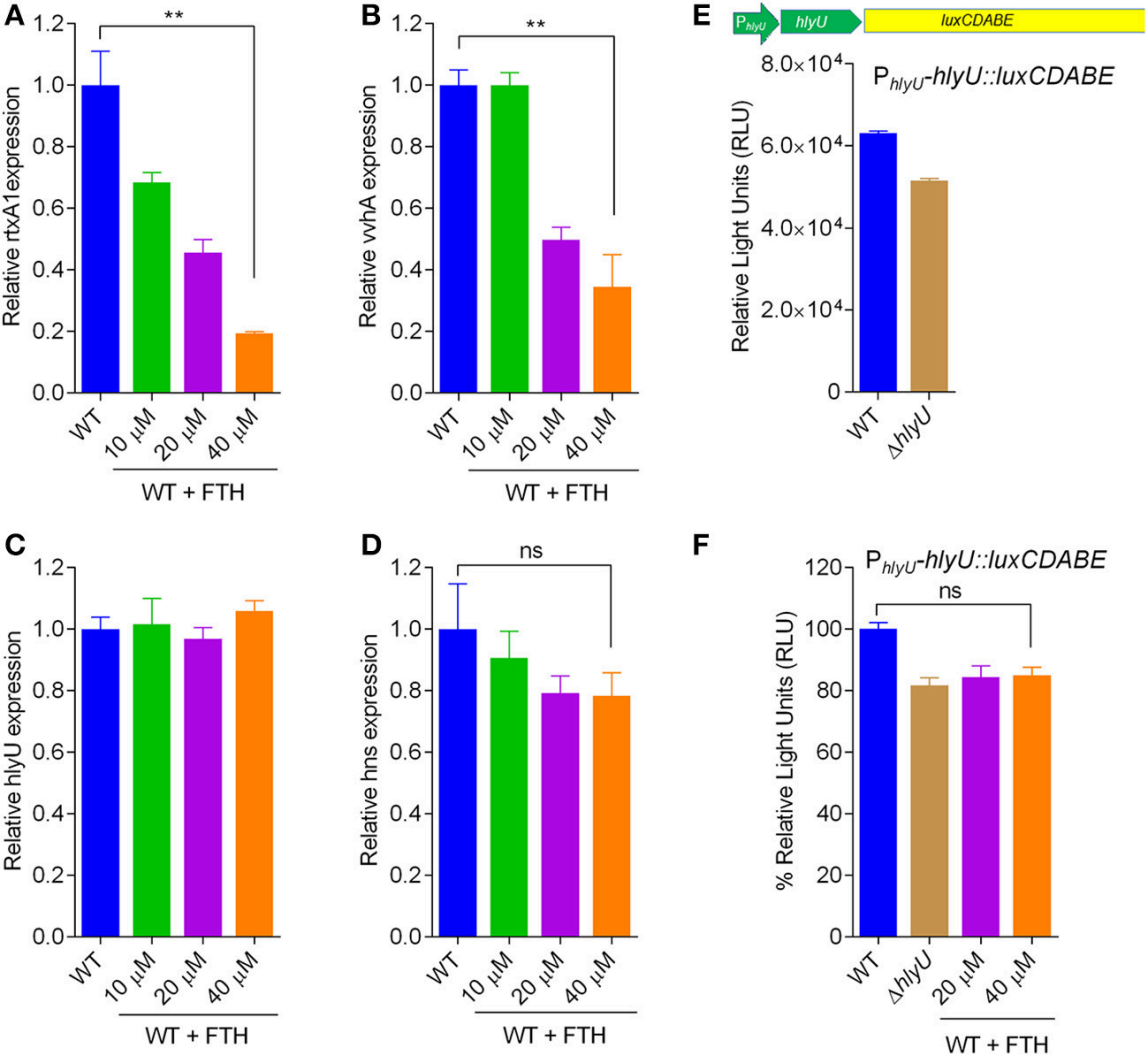
Concentration-dependent expression of genes in the HlyU regulatory network in response to FTH treatment of wild type *V. vulnificus*. The relative expression of genes in response to varying concentrations of FTH is depicted as follows: **(A)** *rtxA1*, encoding the RtxA1 toxin; **(B)** *vvhA*, encoding hemolysin; **(C)** *hlyU*, encoding HlyU transcriptional regulator, and **(D)** *hns*, encoding a global repressor. Respective DMSO controls showed no difference in gene expression of WT sample. **(E)** A transcriptional fusion of P_*hlyU*_-*hlyU*::*luxCDABE* gene showing the level of luminescence in wild type and Δ*hlyU V. vulnificus*; **(F)** Revalidation of *hlyU* transcription with varying concentration of FTH depicts the unchanged level of *hlyU* transcript in wild type *V. vulnificus* harboring the transcriptional fusion P_*hlyU*_-*hlyU*::*luxCDABE*. The percent RLU per unit OD_600_ was calculated based on the RLU values shown in **(E)**.

Although the rationale for the design and development of the reporter strain was based on known transcriptional conduits of HlyU (Figures 1I,II), it has not yet been confirmed whether FTH works by targeting HlyU transcriptional regulator. Therefore, the effect of FTH on the transcription of *rtxA1* and other known genes involved in its regulatory cascade such as *vvhA* and *hns* (Figure 1) were tested using qRT-PCR (Figure 3). As expected, FTH inhibited the transcription of *rtxA1* and *vvhA* genes in a dose-dependent manner (Figures 3A,B). HlyU regulates another virulence determinant phospholipase A_2_ (*plpA*_2_) in *V. vulnificus* which has been recently reported (Jang et al., 2017). The phospholipase A_2_ is essential for host cell lysis and necrotic epithelial cell death. FTH significantly (*P* < 0.05) decreased the *plpA*_2_ gene expression in *V. vulnificus* (Figure S4). The result indicates that FTH targets HlyU function and thereby repress the various virulence determining genes along with toxin encoding genes network.

There was no significant change in the transcription of *hlyU* upon FTH treatment (Figure 3C). Similarly, FTH did not show a significant modulatory effect on the transcription of the *hns* gene when compared to the untreated control (Figure 3D). H-NS is a known repressor of *rtxA1* and *vvhA* toxin encoding genes, thus the alteration in *hns* gene expression may also change toxin gene expression. The consistent *hlyU* transcript level following FTH treatment was further confirmed using a transcriptional fusion of *hlyU* with the promoter-less *luxCDABE* reporter (P_*hlyU*_*_hlyU*::*luxCDABE*) under its native P_*hlyU*_ promoter in WT *V. vulnificus* (Figures 3E,F). Varying concentrations of FTH added to WT *V. vulnificus* possessing the transcriptional fusion construct showed no inhibition in luminescence per unit OD_600_, suggesting unchanged transcriptional levels of the *hlyU* gene. The constant *hlyU* transcript levels observed with and without FTH treatment by both qRT-PCR (Figure 3C) and by transcriptional fusion studies (Figure 3F) supports the hypothesis that FTH does not target upstream regulatory genes in the HlyU transcriptional regulatory network (Figure 1I). In addition, the effect of FTH on the HlyU-regulated gene expression in *V. vulnificus* reporter, Δ*hlyU* knockout reporter and Δ*hlyU* complemented strains was evaluated, wherein Δ*hlyU* harboring the reporter plasmid served as negative control. The FTH concentration dependent inhibition of *rtxA1* and *vvhA* gene-expression in the Δ*hlyU* complemented strain reproducibly confirmed that FTH operates through HlyU transcriptional regulator without disturbing the gene expression of both *hlyU* and *hns* regulators (Figure S6).

### *In vivo* assessment for specific targeting of HlyU using FTH

To affirm that the inhibition of the RtxA1 and VvhA cytotoxins by FTH occurs specifically through HlyU, an 89 bp synthetic promoter (P_*EM*7_) was cloned into the promoter-less *luxCDABE*-containing pBBRMCS2 plasmid and electroporated into WT and Δ*hlyU V. vulnificus*. WT *V. vulnificus* containing P_*EM*7_::*luxCDABE* was used as a non-specific control to assess the specificity of FTH toward HlyU, as HlyU drives the reporter gene expression under the P_*rtxA*1_ promoter (Figure S5A). In a comparative analysis of WT and Δ*hlyU* reporter strains, Δ*hlyU* reporter strain showed only one third the luminescence of the WT reporter strain, re-emphasizing the direct role of HlyU in the regulation of the *rtxA1* toxin gene (Figure S5B). In a comparative assessment of the effect of FTH on the promoter activities of P_*rtxA*1_ (specific) vs. P_*EM*7_ (non-specific) in the presence of native chromosomal HlyU, the wild type reporter strain displayed specific inhibition of luminescence with P_*rtxA*1_ in response to varying concentrations of FTH, but no inhibition was observed with the synthetic promoter P_*EM*7_ tagged with *luxCDABE*. This suggests that FTH specifically targets the HlyU transcriptional regulator, and attenuates the expression of the RtxA1 toxin under ambient bacterial growth conditions (Figure S5C).

### Inhibition of the hemolysis activity of *V. vulnificus* by FTH

**Figure 4:**
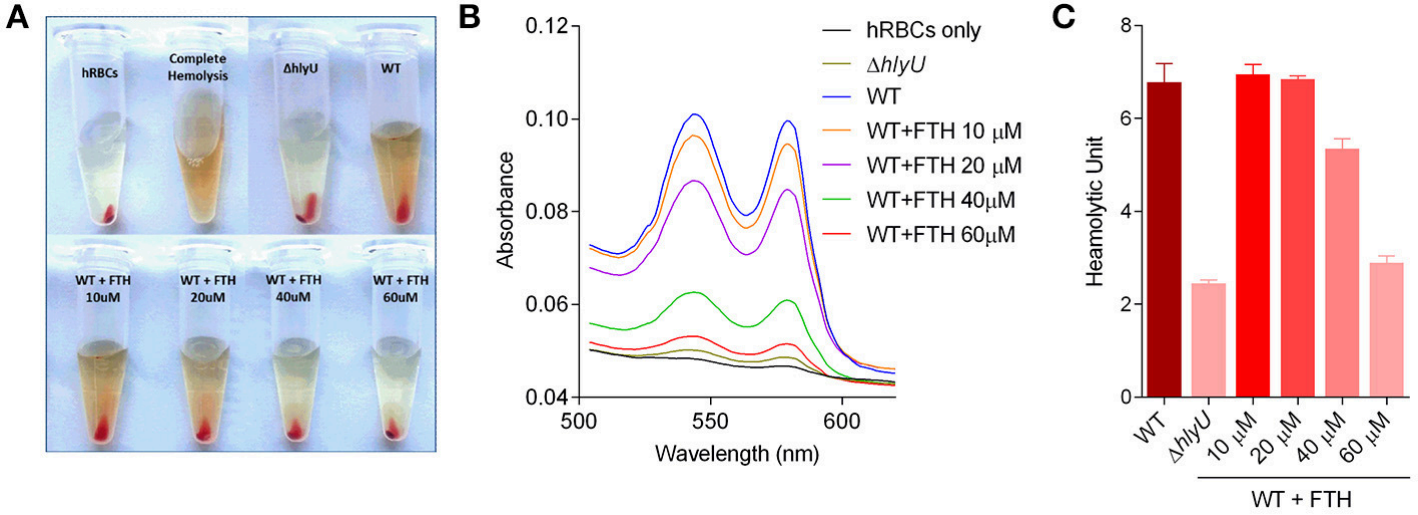
The effect of FTH on the hemolytic activity of *V. vulnificus*. Untreated and FTH-treated (10, 20, 40, and 60 μM) 3 h culture supernatants of WT *V. vulnificus* were used for hemolysis visualization and hemolytic unit calculation (see method for details). **(A)** Visualization of hemolysis by WT *V. vulnificus* in the absence and presence of FTH, and **(B)** the absorption spectra showing the characteristic hemolysis peaks in various treatments. **(C)** Quantitative estimation of hemolysis inhibition activity of FTH expressed as hemolytic unit.

The reduction in *rtxA1* and *vvhA* gene expression upon FTH treatment was further verified by phenotypic assessment of WT *V. vulnificus* on human RBCs (hRBCs) by evaluating the hemolytic activity as hemolytic unit since both RtxA1 and VvhA contribute to hemolytic activity by forming pores in the cell membranes of hRBCs (Kim et al., 2008). The culture supernatant of Δ*hlyU* strains was used as a positive control of hemolysis inhibition. The culture supernatant of WT *V. vulnificus* grown for 3 h in the absence and presence of FTH were treated to hRBCs for hemolytic activity assessment. The inhibition of the hemolytic activity was visualized by the reduction of signature hemolytic peaks in the absorption spectra of FTH treated samples in concentration-dependent manner (Figures 4A,B). The hemolysis of hRBC at 60 μM FTH treatment was found to be negligible and was comparable to the hemolytic units of Δ*hlyU* strain (Figure 4C). These results suggest that the *rtxA1* and *vvhA* gene expression and activity is positively controlled by HlyU. It is noteworthy that the hemolysin activity promotes the early dissemination and growth of bacteria *in vivo*, aiding their spread from the small intestine to other organs and contributing to the development of early pathogenesis during *V. vulnificus* infection (Jeong and Satchell, 2012). Therefore, FTH mediated targeting of the master virulence transcriptional regulator, HlyU, inhibited the production of hemolysin and RtxA1 at the transcriptional level and is expected to reduce the virulence and inhibit the disease progression of *V. vulnificus*.

### Rescuing host cytoskeletal destabilization using FTH

**Figure 5:**
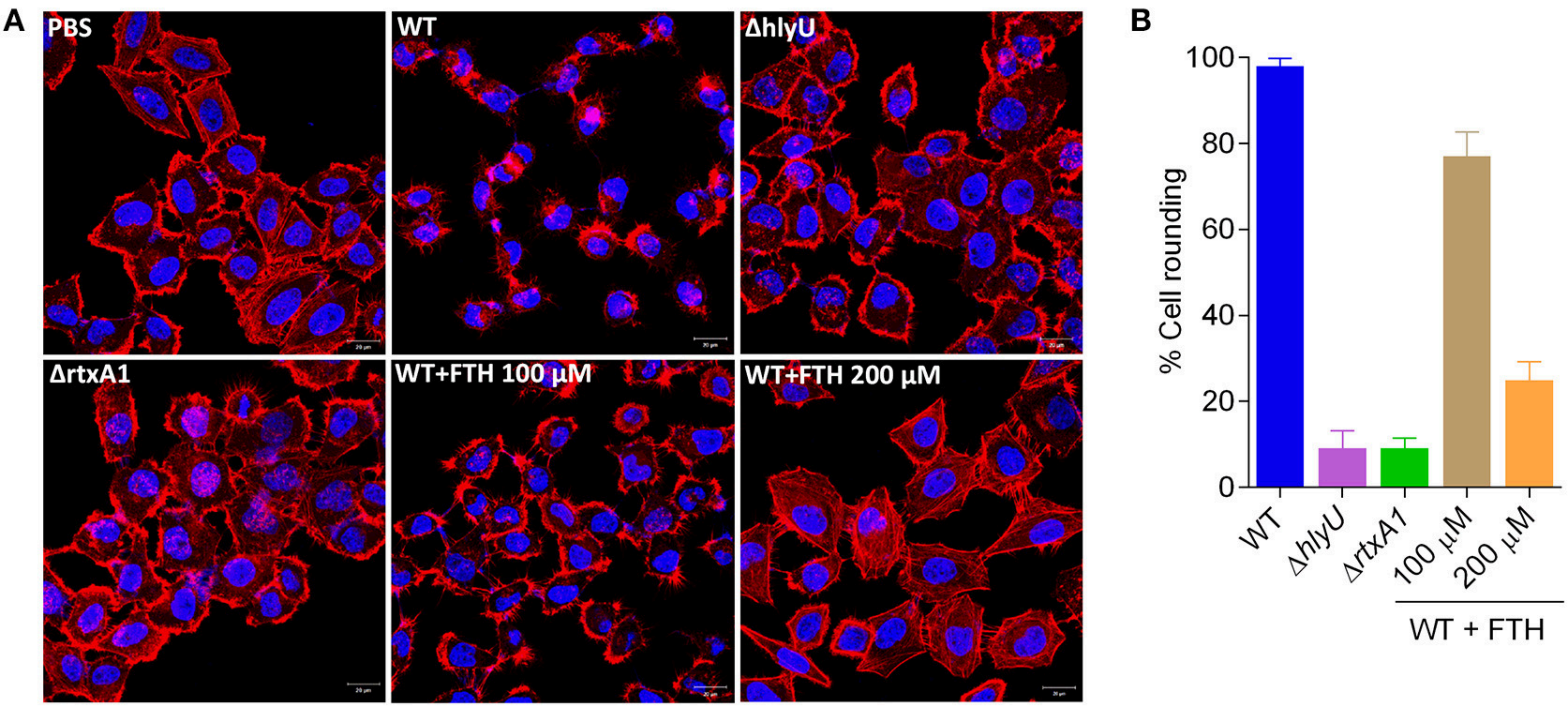
Rescue of cytoskeletal destabilization by FTH treatment using a human cell line. **(A)** HeLa cells were treated with WT *V. vulnificus* with and without FTH (100 and 200 μM), along with Δ*hlyU*, Δ*rtxA1* and PBS controls. *V. vulnificus* control strains (Δ*hlyU* and Δ*rtxA1*) lacking toxins show a discrete cytoskeleton network (red) spread throughout the cytoplasm, while the WT *V. vulnificus* possessing the RtxA1 toxin totally destabilized the cytoskeletal network and cellular morphology. The presence of FTH (200 μM) with WT *V. vulnificus* rescued the cytoskeletal destabilization and protected cells from rounding in a concentration-dependent manner. Scale bar is equal to 20 micron. **(B)** Frequency of cytoskeletal destabilization and consequent rounding of HeLa cells as a measure of RtxA1 activity. A total of 1,000 cells were counted in random microscopic fields for each sample, and percentages were calculated based on round vs. intact shaped cells. FTH treatment inhibited RtxA1 at the transcriptional level. Thus, the cytoskeleton was found to be intact in the FTH-treated wild type sample (normal cell shape at 200 μM) but not in the untreated WT sample (rounded cells).

It has been reported that the expression of *rtxA1* toxin of *V. vulnificus* dramatically increase by contact to host cells in a time-dependent manner. Our results showed transcriptional repression of the *rtxA1* gene by FTH treatment, and thus, it is necessary to further confirm the effect of FTH by assessing the phenotype of the RtxA1 toxin on the host cells. Cytoskeletal destabilization is the hallmark of the RtxA1 toxin, along with plasma membrane blebbing and consequent necrotic cell death by cytolytic activity (Kim et al., 2008). Therefore, RtxA1 level can be estimated by the RtxA1-mediated destabilization of cytoskeleton, which results in the distortion of cell shape tending toward cell rounding. In this study, we monitored the cell rounding by staining the F-actin component of the cytoskeleton in HeLa cells using rhodamine phalloidin. WT *V. vulnificus* triggered more than 95% cell rounding while the FTH treatment (200 μM) merely affected 25 ± 5% host cells compared to 11 ± 4% round-cells in Δ*hlyU* and Δ*rtxA1* mutant controls (Figures 5A,B). We also checked that FTH (100 and 200 μM) has insignificant effect on bacterial CFU of WT *V. vulnificus* in DMEM after an hour of treatment, using the same experimental conditions (Figure S3). FTH-mediated significant (75%) protection of host cells from RtxA1-induced cytoskeletal destabilization and cell-rounding is well represented by the transcriptional inhibition of *rtxA1*, confirming that FTH plays a role in suppressing the HlyU-regulated gene expression of *rtxA1*.

### Avoidance of antivirulence resistance in *V. vulnificus* by FTH

**Figure 6:**
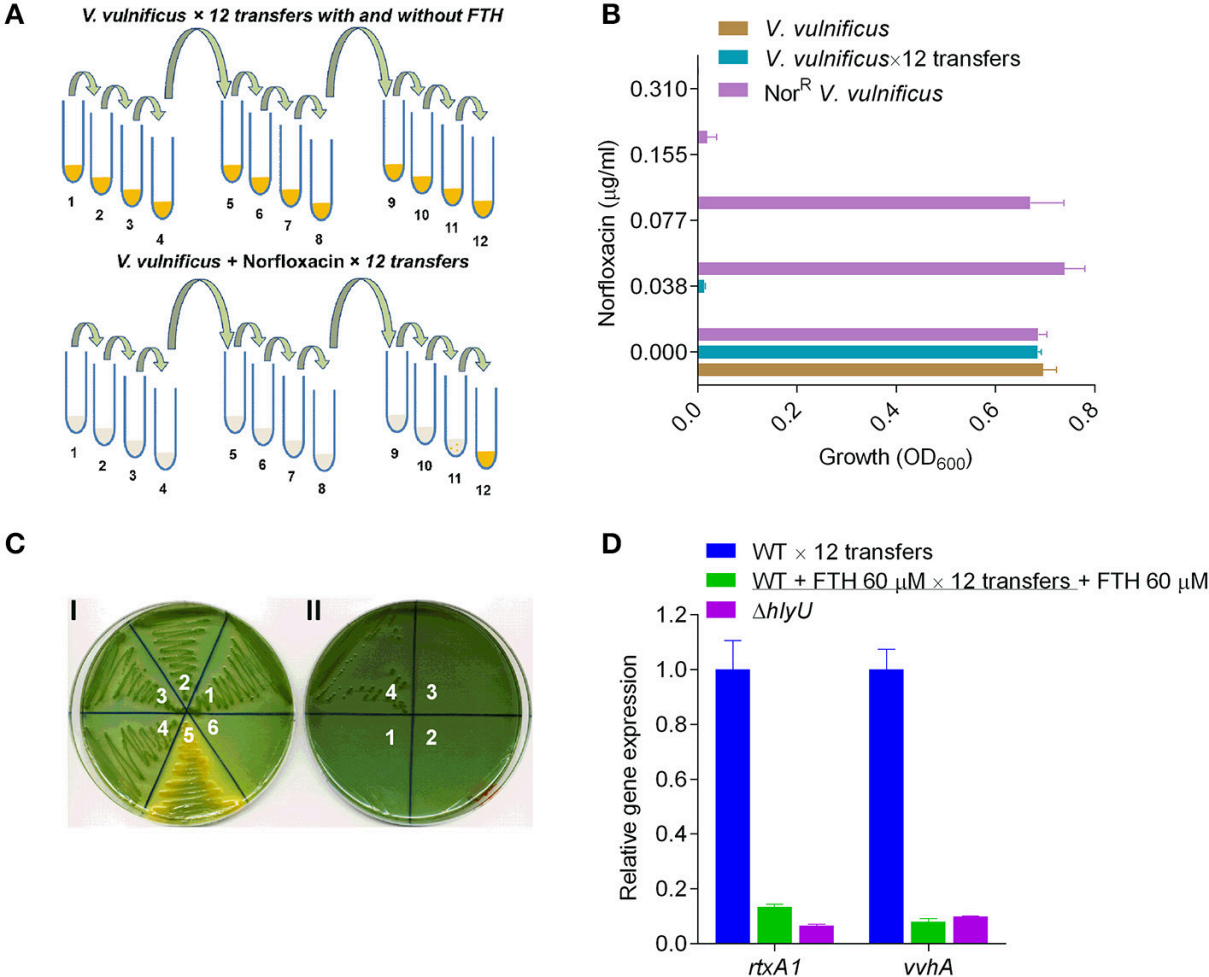
Laboratory-induced adaptive evolution of antimicrobial vs. antivirulence resistance in *V. vulnificus*. **(A)** Schematic diagram showing laboratory-induced adaptive evolution by continuous transferring of *V. vulnificus* exposed to norfloxacin (0.155 μg/ml) or FTH (40 and 60 μM). The numbers below the tubes represent the number of transfers. Yellow color in tubes denotes the growth. **(B)** Evaluation of norfloxacin MIC against non-transferred *V. vulnificus*; untreated *V. vulnificus* transferred 12 times (*V. vulnificus* × 12 transfers) and adaptively evolved, norfloxacin-resistant *V. vulnificus* (Nor^R^ *V. vulnificus*). For MIC calculations, an initial bacterial inoculum of 1 × 10^5^ CFU/ml was used for Nor^R^ *V. vulnificus, V. vulnificus*, and *V. vulnificus* × 12 transfers. Nor^R^ *V. vulnificus* adapted to grow at 0.077 μg/ml, in contrast to the WT *V. vulnificus* control strain without any treatment. This data showed that *V. vulnificus* can evolve and acquire resistance in order to survive sublethal antibacterial concentrations in a very short amount of time. **(C)** Verification of identity of the adaptively evolved, putative Nor^R^ *V. vulnificus* strain. **(I)** Various numbers (1-6) on TCBS agar plate represent the original stock wild type *V. vulnificus* (1), the untreated *V. vulnificus* × 12 transfers (2), FTH-treated *V. vulnificus* (3), the adaptively evolved Nor^R^ *V. vulnificus* (4), a sugar-fermenting *V. alginolyticus* strain acts as a positive control of histochemical plate for sugar fermenting *Vibrio* (5), and *E. coli* DH5α as negative control (6). All the *Vibrio* strains (but not *E. coli* DH5α) grew on the TCBS agar plates. **(II)** Reconfirmation of norfloxacin resistance of adaptively evolved Nor^R^ strain on TCBS agar plate supplemented with 0.155 μg/ml norfloxacin. Growth of the adaptively evolved Nor^R^ strain (4), on TCBS-norfloxacin plate while the controls (1–3) did not show any sign of growth confirms its identity and its evolved norfloxacin resistance in *Vibrio* species. **(D)** The antivirulence resistance of FTH-exposed strains were analyzed by gene expression of *rtxA1* and *vvhA* to verify resistance against FTH. The qRT-PCR results showed the same level of *rtxA1* and *vvhA* transcriptional inhibition in the FTH-exposed strain and in the original stock culture of *V. vulnificus*.

Antimicrobial resistance that arises in various bacterial pathogens during antibiotic-mediated treatment is caused by adaptive evolution and clonal selection due to sustained antibiotic pressure. AMR in *V. vulnificus* appears to be rare. However, a few examples of *V. vulnificus* AMR have been reported recently (Elmahdi et al., 2016). To better understand AMR emergence, norfloxacin was chosen to test laboratory AMR development at a concentration of 0.155 μg/ml. This concentration was adopted by taking the average of a range of reported minimum inhibitory concentration (MIC_90_) values for six *Vibrio* strains (0.063–0.25 μg/ml) (Morris et al., 1985). To examine the adaptive evolution of AMR in the laboratory, wild type *V. vulnificus* was repeatedly transferred with or without 0.155 μg/ml norfloxacin, or with 40 or 60 μM FTH, along with untreated controls. A clumped-particulate suspension, likely *V. vulnificus*, appeared at the eleventh transfer. At the twelfth transfer, a visible turbidity appeared in tubes containing *V. vulnificus* treated with the norfloxacin (0.155 μg/ml) (Figure 6A). The adapted cultures were preserved at twelfth transfer for further analysis. Norfloxacin resistance and the preliminary identity of the adaptively evolved norfloxacin resistant (Nor^R^) strain, along with the untreated cultures, were verified by MIC assessment (Figure 6B) and growth on *Vibrio*-specific TCBS agar plates (Figure 6C). This was followed by streaking, growth, and colony phenotype observation on TCBS agar plates containing norfloxacin (0.155 μg/ml) (Figures 6C I,II). The norfloxacin-adapted Nor^R^ strains could grow on TCBS norfloxacin plates (0.155 μg/ml) but the untreated or FTH-adapted strain did not produce any colonies, even in the inoculation zone (Figure 6C II). The identity of the evolved Nor^R^ strain, the FTH-treated strain, and the −80°C stock cultures were further verified by PCR amplification and sequencing analysis using 16S rRNA gene-specific primers for *V. vulnificus* MO6-24/O (Table S2). Sequence analysis of the amplified 16S rRNA genes demonstrated that the evolved norfloxacin-resistant (Nor^R^) *V. vulnificus* strain and the FTH-treated strain originated from the same *V. vulnificus* strain (MO6-24/O).

We also tested the development of antivirulence resistance in FTH-exposed strains by analyzing *rtxA1* gene expression of WT *V. vulnificus* treated with 60 μM FTH. The qRT-PCR results showed the same level of *rtxA1* transcriptional inhibition in the FTH-adapted strain as in the original stock culture of *V. vulnificus* (Figure 6D). These results suggest that an antivirulence approach can be used to avoid the development of both antimicrobial and antivirulence resistance (Figures 6C,D). Specifically, drug resistance acquired through short-term acclimatization (by metabolic modulation) or through the long-term acquisition and accumulation of mutations is unlikely when using an antivirulence agent of nutraceutical nature.

## Discussion

Antibiotics are certainly one of the most celebrated achievements of human history, and their precise, targeted use has saved millions of patients from deadly infectious diseases. However, the indiscriminate, excessive, and untargeted use of antibiotics has enhanced the rate of AMR emergence. *V. vulnificus* is an emerging, antibiotic-resistant, opportunistic pathogen. Antibiotics such as the quinolones and tetracycline are successfully employed in the treatment of *V. vulnificus* infections. However, the emergence of a higher rate of AMR (Baker-Austin et al., 2009; Baker-Austin and Oliver, 2018) and a lower rate of discovery of new antibiotics not only worsen the prophylaxis and treatment regime but also push back the mankind to the pre-antibiotic era to reconsider alternative approaches to treat AMR infections. Bacteriophages are being used as a specific and targeted alternative therapy to address the AMR strains both in environmental and clinical setting (Cooper et al., 2016; Letchumanan et al., 2016; Yen et al., 2017). However, the “phage-resistance” can also occur at a very high rate which may cause the debility of phage and consequent survival and persistence of bacterial pathogen during treatment (Aminov et al., 2017; Yen et al., 2017). Similarly, probiotics such as *Streptomyces sp*. is known to reduce the *Vibrio* sp. load in aquaculture. Nevertheless, the accumulation of the off-flavor compounds and lateral gene transfer of antibiotics resistance gene are the current limitations associated with probiotics application (Tan et al., 2016). Thus, as an alternative to antibiotics, bacteriophages and probiotics treatment, another anticipated evolution-proof antivirulence approach is being developed to treat AMR bacterial infections. The disarmament of early virulence factors, such as cytotoxins, in *V. vulnificus* at the transcriptional level using an antivirulent small molecule represents an attractive strategy to inhibit both pathogenesis and antibiotic resistance (Figure 1). Early virulence factors responsible for the establishment and dissemination of pathogenesis include RtxA1 and hemolysin (VvhA), which are highly invasive pore-forming toxins (PFTs). Cytotoxins play essential roles in establishing pathogenesis in the host by (**a**) supporting pathogen entry into the host cells (**b**) destabilizing the cytoskeletal network of host cells to inhibit phagocytosis (Lo et al., 2011), and (**c**) evading the innate immune system for rapid dissemination into the bloodstream (Song et al., 2016). The HlyU transcriptional regulator de-represses the expression of cytotoxins; HlyU can therefore be targeted using small molecules to inhibit *V. vulnificus* pathogenesis by inhibiting expression of key cytotoxins.

The reporter platform for the inhibitor-screening was developed using reverse chemical genetics in the target microbe, *V. vulnificus*, based on the HlyU regulatory cascade (Figures 1I,II). Specifically, the native chromosomal HlyU binds to the P_*rtxA*1_ promoter tagged with a promoter-less *luxCDABE* in the reporter plasmid. This plasmid-based reporter system showed a background signal even in the absence of HlyU, presumably due to low leaky expression and multiple copy number. The background luminescence in Δ*hlyU V. vulnificus* was therefore used as the control background signal (Figure S1). By screening chemicals using the luminescence reporter system, we identified fursultiamine hydrochloride (FTH) as a potent small molecule inhibitor (Figure 2) that decreased the expression of cytotoxins under the control of HlyU (Figures 1III,IV). FTH is a vitamin B1 derivative or synthetic thiamine analog, originally used to treat thiamine deficiency (Lonsdale, 2004). There is a single report of an inhibitor of *rtxA1* transcription (Kim et al., 2010). In this report, resveratrol was identified from a cell-viability assay as a modulator of host-microbe interactions in terms of adhesion, motility, and consequent cytotoxicity (Kim et al., 2010), but its specific regulatory cascade has not been identified. However, in the current study, we identified FTH as an inhibitor of HlyU transcriptional regulator from *rtxA1* transcription targeted—reporter platform, and validated its inhibition activity by checking the expression of HlyU downstream genes and global repressor genes (Figure 3). In addition, we further verified that the transcription of *hlyU* remained unchanged by FTH by examining a transcriptional fusion of *hlyU* (with its native promoter) to *luxCDABE* (Figures 3E,F). The results with transcriptional fusion indicated that FTH-mediated inhibition of luminescence in the WT *V. vulnificus* reporter strain was not due to the inhibition of any known or unknown upstream gene/regulator (Figure 1I).

The revalidation of FTH target using forward chemical genetics showed that FTH targeted HlyU, wherein the specific P_*rtxA*1_ promoter and a non-specific, synthetic P_*EM*7_ promoter were used to drive the expression of *luxCDABE* reporter operon. The specific inhibition of luminescence by FTH was concentration-dependent and only occurred with the P_rtxA1_ (and not the P_*EM*7_) promoter. Thus, several lines of experimental evidence distinctly suggest that FTH inhibits the expression of virulence factors through HlyU (Figure S5). Although FTH targets HlyU transcriptional regulator, the precise inhibitory mode of action of FTH on HlyU protein function remains unexplored and should be considered for future study. Hemolytic activity due to RtxA1 and VvhA was reduced upon FTH treatment and was comparable to the activity of Δ*hlyU V. vulnificus* (Figure 4). This finding is consistent with the transcriptional data inhibiting the expression of these toxins (Figure 3). RtxA1, or multifunctional autoprocessing repeats-in-toxin (MARTX) is known to be secreted through type I secretion system (Kim et al., 2013). FTH significantly reduced the cytoskeleton destabilization activity of RtxA1 on host cells (Figure 5). Cytoskeleton is crucial for phagocytosis (Swanson and Baer, 1995) and RtxA1 is known to inhibit phagocytosis which eventually helps *V. vulnificus* survival by evading host innate immune system (Lo et al., 2011). Taken together, the treatment of antivirulence FTH molecule targeting the HlyU, disarmed major potent pore forming toxins (RtxA1 and VvhA) and transformed them into a strain with reduced virulence (Figure 1V; Figures 4, 5).

The development of evolution proof antivirulence drug against pathogenic bacteria is an emerging challenge which can be alleviated by designing virulence factor specific genetically engineered screening platform and the careful examination of the small molecule leads for characteristics of an antivirulence drug, which has elegantly been opined and explained (Allen et al., 2014). In this context, the laboratory adaptive evolution of *V. vulnificus* against norfloxacin treatment demonstrates the rapid and enormous capability of this pathogen to acclimatize against antibiotic stress (Figure 6). Interestingly, continuous exposure of *V. vulnificus* to the antivirulence compound FTH did not cause the development of antivirulence resistance (Figure 1VI; Figure 6) presumably due to its nontoxicity to both host and pathogen and nutraceutical nature. However, there is a frequent trade-off between pharmaceutical and nutraceutical molecules for potency and toxicity levels. To support the *in vitro* data, we attempted to validate the FTH efficacy as an antivirulent drug by establishing a simple yet strong immune system possessing *Galleria mellonella* (greater wax moth) infection model (Loh et al., 2013) for the first time, for *V. vulnificus* (Figure S7). FTH showed marginally higher protection (~30% survival) of the wax moth larvae against *V. vulnificus* infections than that of FTH untreated infection group (10% survival) (Figure S8). Unfortunately, we found that the FTH-mediated protection data is inconsistent with a high level of variability in 3 independent experiments presumably due to *in vivo* drug instability in wax moth larvae as reported earlier in mice model (Fung et al., 2013). The instability of FTH may be alleviated by generating a wide range of FTH derivatives by protecting its antivirulence activities in future studies.

Despite the *in vivo* instability issue, FTH has several advantages as an antivirulence agent, such as (**a**) enhanced bioavailability due to its reduced absorption barrier (**b**) a high IC_50_ that poses no toxicity to the host (**c**) effective targeting of virulence transcription factor, HlyU (**d**) transcriptional inhibition of highly invasive cytotoxins, and (**e**) avoidance of AMR development during antivirulence strategy to tackle *V. vulnificus* infections presumably due to its nutraceutical nature and low selection pressure (Figures 1V,VI). In conclusion, FTH is a potential antivirulence agent that may be useful in combating *V. vulnificus* pathogenesis by inhibiting the transcriptional network of cytotoxins without causing the emergence of antimicrobial and antivirulence resistance. Our results also suggest that an antivirulence strategy targeting the expression of virulence factors regulated by HlyU might be a promising approach for the treatment of infectious disease caused by *V. vulnificus*.

## Author contributions

SI and AC performed the experiments. SI and AC wrote the manuscript with the support of KK. The experiments were designed and performed mainly under the supervision of AC and KK.

### Conflict of interest statement

The authors declare that the research was conducted in the absence of any commercial or financial relationships that could be construed as a potential conflict of interest. The reviewer YM and handling editor declared their shared affiliation.
