# Supplementary material for: Identification and Validation of an Antivirulence Agent Targeting HlyU-Regulated Virulence in Vibrio vulnificus
Source: Front Cell Infect Microbiol. 2018 May 11;8:152. doi: 10.3389/fcimb.2018.00152 (PMC5958221; doi:10.3389/fcimb.2018.00152)
Supplement: Supplementary file 1 [file Data_Sheet_1.docx]

**Supporting Information**

**Identification and validation of an antivirulence agent targeting HlyU-regulated virulence in *Vibrio vulnificus***

**RUNNING TITLE**

Development of an antivirulence agent against *V. vulnificus*

**AUTHORS**

Saba Imdad, Akhilesh K. Chaurasia* and Kyeong Kyu Kim*

Department of Molecular Cell Biology, Samsung Medical Center, Sungkyunkwan University School of Medicine, Suwon, 16419, Korea

* Corresponding authors:

E-mail: [chaurasia@skku.edu](mailto:chaurasia@skku.edu) or [kyeongkyu@skku.edu](mailto:kyeongkyu@skku.edu)

Tel: 82-31-299-6136

Fax: 82-31-299-6159

**Contents:**

**Figures**

**Figure S1.**

Optimization of *V. vulnificus* reporter strain for luminescence signal with and without HlyU.

**Figure S2.**

Confirmation of non-antibiotic activity of FTH against *V. vulnificus.*

**Figure S3.**

Effect of high dose of FTH on *V. vulnificus.*

**Figure S4.**

Inhibition of expression of another HlyU-regulated virulence gene, *plpA_2_* by FTH.

**Figure S5.**

*In vivo* assessment for specific targeting of HlyU using FTH.

**Figure S6.**

Effect of FTH on the gene expression of ∆*hlyU* complementation strain.

**Figure S7.**

Establishment of *Galleria mellonella* infection model for *V. vulnificus*.

**Figure S8.**

*In vivo* efficacy of FTH using *G. mellonella* (wax moth) infection model.

**Supplementary Tables**

**Table S1.** Bacterial strains and plasmids used in the study

**Table S2.** Oligonucleotides primers used in this study

**
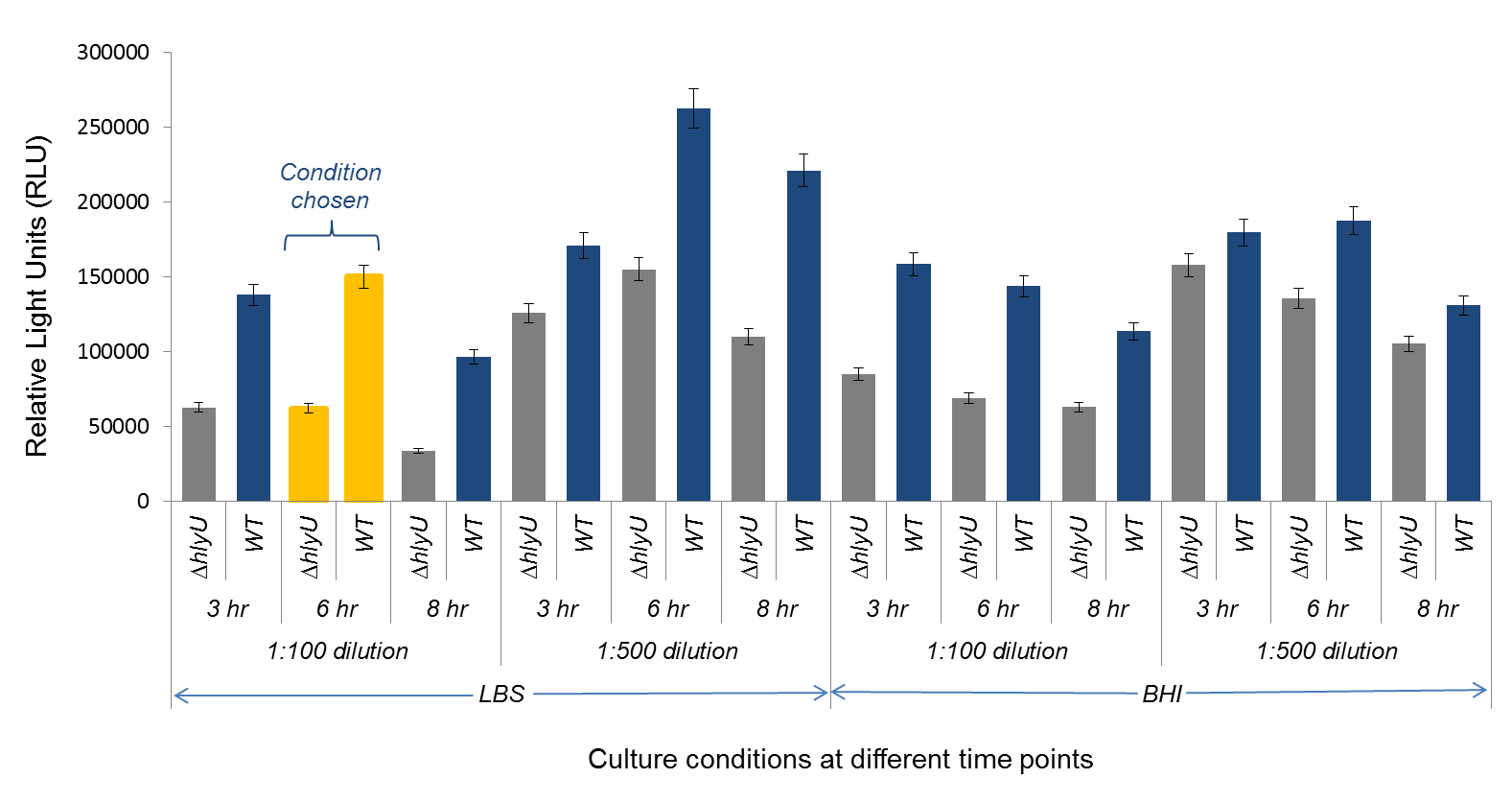
**

**Figure S1**. **Optimization of *V. vulnificus* reporter strain for luminescence signal with and without HlyU.** LBS and brain heart infusion (BHI) -grown overnight cultures of wild type (WT) and ∆*hlyU* strains of *V. vulnificus* carrying the reporter construct pBBRMCS2_P*_rtxA1_*::*luxCDABE* were diluted by 1:100 and 1:500 in microtiter plates. The plates were incubated for the specified time intervals (3 h, 6 h and 8 h) in an orbital shaker at 37°C. Luminescence and optical density at 600 nm (OD_600_) were recorded at each time point using a microplate reader (Tecan Infinite M200, Switzerland). The relative luminescence unit (RLU) per unit OD_600_ represents regulation of the P*_rtxA1_* promoter in the presence or absence of the HlyU transcriptional regulator. The conditions chosen were based on paired minimum and maximum luminescence signals in WT and ∆*hlyU* reporter strains. Condition chosen for inhibitor screening included: 1:100 inoculum dilution ratio, growth in LBS medium for 6 h, and incubation at 37°C in orbital shaking culture conditions at 220 rpm.

**
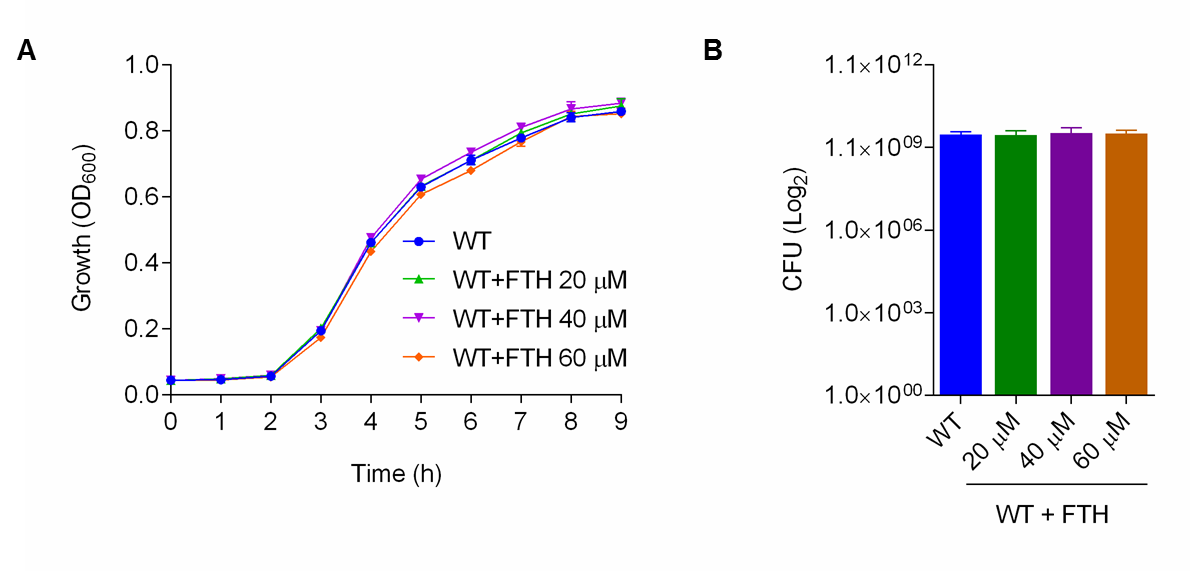
**

**Figure S2**. **Confirmation of non-antibiotic activity of FTH against *V. vulnificus*. (A)** Time-dependent measurement of optical density at 600 nm (OD_600_) of WT *V. vulnificus* with DMSO vehicle (maximum DMSO concentration = 60 µM, plotted) and with varying concentrations of FTH (20, 40, and 60 μM) in LBS. **(B)** Colony forming units (CFU) of *V. vulnificus* with and without FTH treatment after 9 h of incubation in LBS. FTH possessed no antibacterial activity against WT *V. vulnificus* at the tested concentrations, as there was no growth retardation observed by optical density and CFU assessment.





**B**

**A**

**Figure S3**. **Effect of high dose of FTH on *V. vulnificus.*** (**A**) The log phase grown WT *V. vulnificus* culture was adjusted to obtain 10^6^ equivalent cells after washing with PBS. The bacterial cells were incubated in DMEM medium with and without FTH (100 and 200 µM) for an hour. The bacterial cells were harvested and washed with PBS before plating on LBS agar plates to determine the CFU. The CFU difference between WT and WT treated with highest tested FTH concentration (200 µM) was comparable. (**B**) Time dependent growth response of WT *V. vulnificus* in LBS media with and without FTH treatment (100 and 200 µM) in LBS.

**

**

**Figure S4**. **Inhibition of expression of another HlyU-regulated virulence gene, *plpA_2_* by FTH.** WT *V. vulnificus* reporter strain was treated with varying non-inhibitory concentrations of FTH for 9 h in 48-well plate and RNA was isolated to prepare cDNA for subsequent qRT-PCR analysis of *plpA_2_* gene encoding phospholipase A_2_ which is essential for *V. vulnificus* virulence. plpA_2_ gene expression was decreased in FTH treated sample (200 µM) as compared to untreated WT reporter strain, similar to ∆*hlyU* reporter control strain.


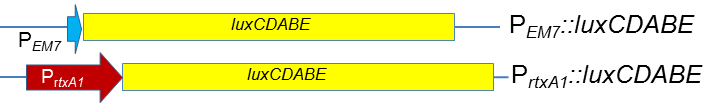


**A**

**B**





**C**

**Figure S5.** ***In vivo* assessment for specific targeting of HlyU using FTH (A)** The constructs showing the P*_rtxA1_* (*rtxA1* promoter: 754 bp) and P*_EM7_* (Synthetic promoter: 89 bp) cloned into the promoter-less *luxCDABE*-containing pBBRMCS2 plasmid*.* The experiment was conducted with 1% bacterial inoculum diluted from 12 h overnight grown cultures (from freshly streaked colonies on plate), and luminescence data were obtained after 2 h of incubation. The relative arbitrary units shown were used to calculate percent RLU inhibition. DMSO controls (not shown) did not show any significant effect on RLU. To calculate FTH percent RLU inhibition, respective DMSO controls were used as 100%. **(B)** Comparison of HlyU-regulated expression of *luxCDABE* in P*_rtxA1_*::*luxCDABE* and P*_EM7_*::*luxCDABE*. **(C)** The effect of FTH on the promoter activities of P*_rtxA1_* (specific) and P*_EM7_* (non-specific) in relation to HlyU. The specific inhibition of luminescence with P*_rtxA1_* in response to varying concentrations of FTH (compared to no inhibition with the synthetic promoter P*_EM7_*) suggests that FTH specifically targets HlyU *in vivo*.

**

**

**B**

**D**

**A**

**C**

**Figure S6. Effect of FTH on the gene expression of ∆*hlyU* complementation strain**. The complementation strain of ∆*hlyU* mutant was created by cloning *hlyU* gene with its native promoter, P*_hlyU_* in promoter-less vector *pBBRMCS2::luxCDABE.* WT *V. vulnificus* reporter, ∆*hlyU* reporter control and ∆*hlyU* complementation strains were treated with varying concentrations of FTH for 9 h in 48-well plate and cDNA was prepared from RNA for qRT-PCR analysis of the genes in the *hlyU* regulatory network. **(A)** *rtxA1* gene expression is restored in ∆*hlyU* complementation strain and upon treatment of FTH, it is significantly decreased with the dose; **(B)** *vvhA*, encoding hemolysin, gene expression showed a similar trend as *rtxA1*; (**C**) *hlyU* and (**D**) *hns,* gene expression was negligibly altered upon FTH treatment.

**Establishment of *Galleria mellonella* infection model for *V. vulnificus***

*G. mellonella,* also known as the greater wax moth is a popular model for studying infection and therapeutic efficacy of new antibiotic drugs because of its strong immune system, ease in handling and no ethical issues (Cytrynska et al., 2007; Loh et al., 2013). The infection model for studying *V. vulnificus* pathogenicity and *in vivo* drug efficacy assessment was established for the first time for *V. vulnificus*. Larvae of *G. mellonella* worms were purchased from a local vendor ensuring axenic conditions (<http://sworm.kr/main/index>) and were maintained in the dark at room temperature. The larvae were used within one week of purchase. The larvae selected for experiments weighed 200±30 mg and belonged to the last instar stage. The overnight-grown *V. vulnificus* strains were sub-cultured at 0.3% inoculum for 6-7 h. The bacterial cells were washed and resuspended in 0.9% saline (20 μl) for the intrahemocoelic infection of *G. mellonella* larvae through the left posterior proleg. The inhibitor (20 μl) was administered after one hour of infection *via* the right posterior proleg (Desbois and Coote, 2011). Three concentrations of WT and ∆*hlyU* *V. vulnificus* (*i.e.* 1× 10^7^, 1×10^5^, and 1×10^3^ cells) were employed to study the role of HlyU in the pathogenesis of *V. vulnificus*. After dose optimization, 200-300 CFU was used for *in vivo* validation of the antivirulence activity of FTH. The survival of larvae was monitored for a period of 60 h for standardization and 40 h for the survival assay. *In vivo* experiments were performed with ten larvae per group. Saline (0.9% NaCl) was used as a control to observe the effect of injection trauma. A ‘no manipulation’ control was kept in each experiment.

**
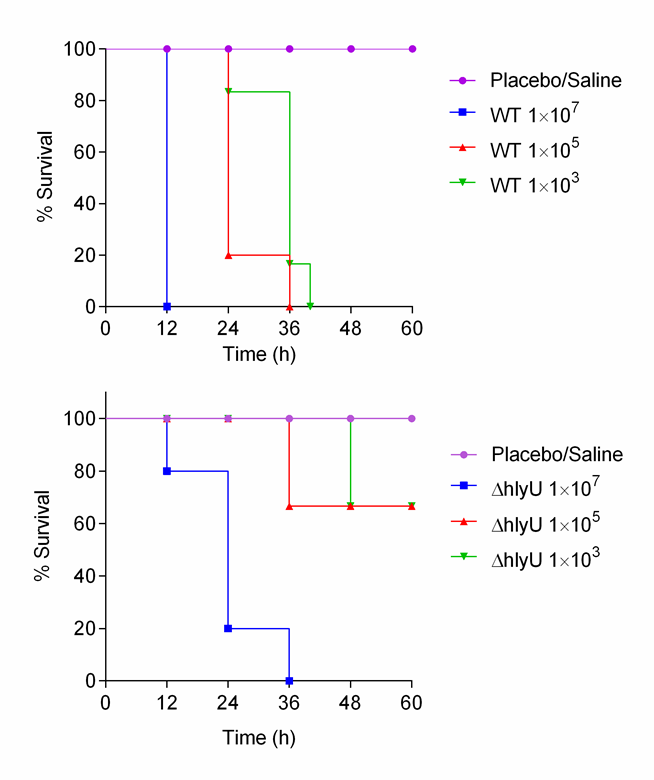
**

**A**

**B**

**Figure S7. Establishment of *Galleria mellonella* infection model for *V. vulnificus*.** Kaplan–Meier survival curves of wax moth larvae at varying *V. vulnificus* cell number treated in 20 μL to the left posterior proleg. Survival was monitored up to 60 h for both the strains **(A**) WT *V. vulnificus* treated larvae and (**B**) ∆*hlyU* *V. vulnificus* treated larvae.

***In vivo* efficacy of FTH using *G. mellonella* (wax moth) infection model.**

FTH effectively inhibited the virulence factors regulated by HlyU when tested under *in vitro* conditions. FTH at the concentration of 67.5 mg/kg, which is 8 times less than its reported LD50 in mice (540 mg/kg) showed marginally higher protection (~30% survival) of the wax moth larvae against *V. vulnificus* infections than that of FTH untreated infection group (10% survival) (**Figure S8**). Fursultiamine is known to convert to thiamine *in vivo*, as FTH compound was originally formulated to increase the absorption barrier of thiamine and to overcome the thiamine deficiency in individuals. Presumably, due to his reason, FTH *in vivo* data using wax moth larvae model showed inconsistency, as reported earlier (Fung et al., 2013).


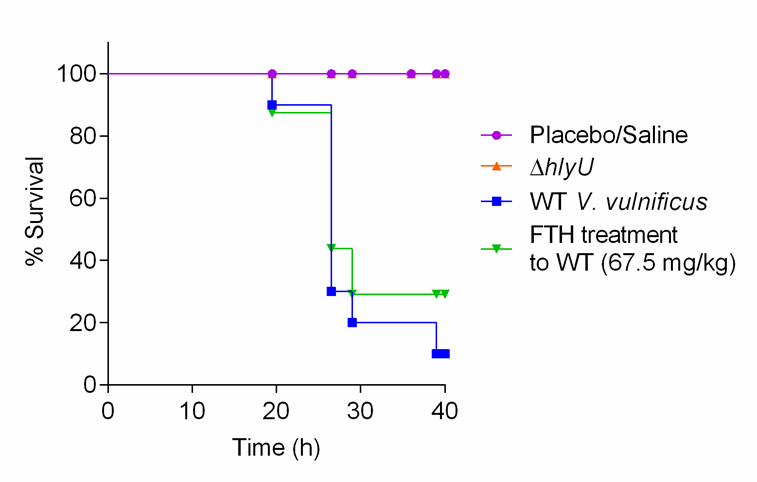


**Figure S8. *In vivo* efficacy of FTH using *G. mellonella* (wax moth) infection model.** Kaplan–Meier survival curves of wax moth larvae using wild type *V. vulnificus* with or without FTH wherein the deletion mutant ∆*hlyU* *V. vulnificus* and saline placebo treated wax moth larvae served as controls for HlyU mediated bacterial virulence inhibition and FTH carrier controls, respectively.

**Supplementary Tables**

**Table S1. Bacterial Strains and plasmids used in the study**

**Designation Relevant Description/Genotype Source**

***Plasmids***

pBBRMCS2::*luxCDABE* Wide host-range vector pBBRMCS2 with promoterless-*luxCDABE*, Cm^R^ (Lenz et al., 2004)

pGEN-*luxCDABE* Erythromycin promoter (P*_EM7_*) containing *luxCDABE* operon, Amp^R^ (Lane et al., 2007) pBBRMCS2_P*_rtxA1_::luxCDABE* pBBRMCS2::*luxCDABE* with *rtxA1* promoter (P*_rtxA1_*), Cm^R^ This study

pBBRMCS2_P*_EM7_::luxCDABE* pBBRMCS2::*lux* with erythromycin promoter (P*_EM7_*), Cm^R^ This study

pBBRMCS2_P*_hlyU_*_*hlyU::luxCDABE* pBBRMCS2::*lux* with *hlyU* with its native promoter (P*_hlyU__hlyU*), Cm^R^ This study

**Bacterial Strains**

*Vibrio vulnificus*

MO6-24/O (WT) Clinical and virulent isolate, wild type (Wright et al., 1990)

WT_pBBRMCS2::*luxCDABE* WT with pBBRMCS2::*luxCDABE*  This study

WT_P*_rtxA1_::luxCDABE* WT with pBBRMCS2_P*_rtxA1_::luxCDABE*  This study

WT_P*_EM7_*::*luxCDABE* WT with pBBRMCS2_P*_EM7_::luxCDABE* This study

WT_P*_hlyU__hlyU*-*luxCDABE* WT with pBBRMCS2_P*_hlyU_*_*hlyU::luxCDABE* This study

∆*hlyU* (ZW141) WT with *hlyU* gene deletion, (Jang et al., 2017)

∆*hlyU*+ pBBRMCS2::*luxCDABE* ∆*hlyU* with pBBRMCS2::*luxCDABE* This study

∆*hlyU*_P*_rtxA1_::luxCDABE* ∆*hlyU* with pBBRMCS2_P*_rtxA1_::luxCDABE* (used as background control) This study

∆*hlyU*_P*_EM7_*::*luxCDABE* ∆*hlyU* with pBBRMCS2_P*_EM7_::luxCDABE* This study

∆*hlyU*_P*_hlyU__hlyU*-*luxCDABE* ∆*hlyU* with pBBRMCS2_P*_hlyU_*_*hlyU::luxCDABE* (also used as ∆*hlyU*

complementation strain) This study

*∆rtxA1* (MW064) WT with *∆rtxA1* gene deletion*,* Km^R^ (Lee et al., 2007)

*E. coli* DH5α F^–^ Φ80*lac*ZΔM15 Δ(*lac*ZYA-*arg*F) U169 *rec*A1 *end*A1

*hsd*R17 (rK^–^, mK^+^) *phoA supE44 λ– thi-1 gyrA96 relA1* Lab stock

**Table S2. Oligonucleotide primers used in this study**

**Purpose Primer Name Sequence**

**Cloning** P*_rtxA1_*-F GTGAATGAGCTCGAATCAAATAAAATGGCGGGTG (SacI)

P*_rtxA1_*-R TCAATGACTAGTTATTTTTTTGATCCTGGCCTAC (SpeI)

P*_EM7_*-F ACCATAGAGCTCTTAAACCCATGGACGTGTTG (BamHI)

P*_EM7_*-R ACGTATCCTCCAAGCCTGAATTC

P*_hlyU__hlyU*-F ACCATAGAGCTCAACCGTTCCCTTGAATACCC (SacI)

P*_hlyU__hlyU*-R AACAAAGGATCCTTATTCTTCGCAATAAAGAC (BamHI)

**qRT-PCR**  *rtxA1*-F GATGGTTACAAAGCCGATAC

*rtxA1*-R TCTGGGTTATCAAGCAGAAT

*vvhA*-F AGACTATCGCATCAACAACC

*vvhA*-R AAACGTCATAGTTCGGTTTG

*hlyU*-F TTCTGCTAAAGCTGTCGTATT

*hlyU*-R AAACCGTTTGTGCTTCTTTA

*hns*-F GAACAAATTGCTAAAGATGGT

*hns*-R GATTTACCCGCATCTAATTG

*plpA_2_*-F ATGGATAATTTGCAGCACTT

*plpA_2_*-R AGCATATTGCTTTGATGAGC

*gyrB*-F TCAGTTTCTGTTAGCGATGA

*gyrB*-R ATCGTCAACAGCACTTTTTC

***WT MO6-24/O* identification** *16S rRNA*-F AGAGTTTGATCATGGCTCAG

*16S rRNA*-R TAAGGAGGTGATCCAGCGC

The underlined nucleotides represent the restiction endonuclease sites shown in bracket.

**References**

Cytrynska, M., Mak, P., Zdybicka-Barabas, A., Suder, P., and Jakubowicz, T. (2007). Purification and characterization of eight peptides from *Galleria mellonella* immune hemolymph. *Peptides* 28**,** 533-546. doi: 10.1016/j.peptides.2006.11.010

Desbois, A.P., and Coote, P.J. (2011). Wax moth larva (*Galleria mellonella*): an *in vivo* model for assessing the efficacy of antistaphylococcal agents. *J Antimicrob Chemother* 66**,** 1785-1790. doi: 10.1093/jac/dkr198

Fung, E., P. Sugianto, J. Hsu, R. Damoiseaux, T. Ganz and E. Nemeth (2013). "High-throughput screening of small molecules identifies hepcidin antagonists." *Mol Pharmacol* **83**(3): 681-690

Jang, K.K., Lee, Z.W., Kim, B., Jung, Y.H., Han, H.J., Kim, M.H., Kim, B.S., and Choi, S.H. (2017). Identification and characterization of *Vibrio vulnificus plpA* encoding a phospholipase A2 essential for pathogenesis. *J Biol Chem*. 292(41)**,** 17129-17143. doi: 10.1074/jbc.M117.791657.Lane, M.C., Alteri, C.J., Smith, S.N., and Mobley, H.L. (2007). Expression of flagella is coincident with uropathogenic *Escherichia coli* ascension to the upper urinary tract. *Proc Natl Acad Sci U S A* 104**,** 16669-16674. doi: 10.1073/pnas.0607898104

Lee, J.H., Kim, M.W., Kim, B.S., Kim, S.M., Lee, B.C., Kim, T.S., and Choi, S.H. (2007). Identification and characterization of the *Vibrio vulnificus* *rtxA* essential for cytotoxicity *in vitro* and virulence in mice. *J Microbiol* 45**,** 146-152

Lenz, D.H., Mok, K.C., Lilley, B.N., Kulkarni, R.V., Wingreen, N.S., and Bassler, B.L. (2004).

The small RNA chaperone Hfq and multiple small RNAs control quorum sensing in *Vibrio harveyi* and *Vibrio cholerae.* *Cell* 118**,** 69-82. doi: https://doi. org/10.1016/j.cell.2004.06.009

Loh, J.M., Adenwalla, N., Wiles, S., and Proft, T. (2013). *Galleria mellonella* larvae as an infection model for group A *Streptococcus.* *Virulence* 4**,** 419-428. doi: 10.4161/viru.24930

Wright, A.C., Simpson, L.M., Oliver, J.D., and Morris, J.G., Jr. (1990). Phenotypic evaluation of acapsular transposon mutants of V*ibrio vulnificus*. *Infect Immun* 58**,** 1769-1773
